# Health Tract, No. 219

**Published:** 1865

**Authors:** 


### NUTRITIOUSNESS OF FOOD.

The following table from authentic sources shows the ascertained per centage of nutriment in the common articles of table consumption. Boiled rice being the easiest of digestion, because the quickest, is marked ten ; boiled cabbage is two ; roast pork, boiled tendon, and beef suet requiring five and a half hours to be digested, would be one, or the lowest grade of digestibility. One important practical bearing of the table is that the most nutritious food should be eaten, as boiled rice, when the bowels are loose; but when constipated, that which has most waste should be eaten, as boiled turnips, because the more waste the greater is the accumulation of this waste in the lower bowel, which acts in proportion as it is distended by such accumulation.

| Kind of Food. | Preparation. | Per Cent of Nutriment. | Time of Digestion. H. M. | Ease of Digestion. | Remarks. |
|---|---|---|---|---|---|
| Almonds,........... | Raw, | 66 | .. | | |
| Apples,............. | " | 10 | 1.30 | 5 | Sweet and mellow. |
| Apricots, .......... | " | 26 | . | . | |
| Barley,............. | Boiled, | 92 | 2.00 | 5 | |
| Beans, dry,........ | " | 87 | 2.30 | 4 | |
| Beef,............... | Roast, | 26 | 3.30 | 3 | Fresh, lean, rare, broiled, digests in three hours. |
| | | .. | .. | . | |
| Blood,.............. | | 22 | .. | | |
| Bread, ............. | Baked, | 80 | 3.30 | 3 | |
| Cabbage,........... | Boiled, | 7 | 4.30 | 2 | |
| Carrots,............ | " | 10 | 3.15 | 3 | |
| Cherries,.... ..... | Raw, | 25 | 2.00 | 5 | |
| Chickens, . ....... | Fricasseed, | 27 | 2.45 | 4 | |
| Codfish, ............ | Boiled, | 21 | 2.00 | 5 | |
| Cucumbers,......... | Raw, | 2 | .. | | |
| Eggs,............... | Whipped, | 13 | 1.30 | 7 | |
| Flour, bolted,...... | In bread, | 21 | .. | . | |
| Flour, unbolted,.... | " | 35 | .. | . | |
| Gooseberries,....... | Raw, | 19 | 2.00 | 6 | |
| Grapes, ............ | " | 27 | 2.30 | 6 | |
| Haddock,........... | Boiled, | 18 | 2.30 | 4 | |
| Melons, ... ....... | Raw, | 3 | 2.00 | 5 | |
| Milk,............... | " | 7 | 2.15 | 5 | |
| Mutton,............ | Roast, | 30 | 3.15 | 3 | |
| Oatmeal, .......... | Baked. | 74 | 3.30 | 3 | |
| Oils, ............... | Raw, | 96 | 3.30 | 3 | |
| Peas, dry,......... | Boiled, | 93 | 2.30 | 4 | |
| Peaches,........... | Raw, | 20 | 2.00 | 4 | |
| Pears, ............. | " | 10 | 3.30 | 6 | |
| Plums,............. | " | 29 | 2.30 | 4 | |
| Pork, .............. | Roast, | 21 | 5.15 | 2 | |
| Potatoes,....... ... | Boiled, | 13 | 2.30 | 4 | |
| Rice,............... | " | 83 | 1.00 | 10 | |
| Rye flour,.......... | Baked, | 79 | 3.30 | 3 | |
| Sole, ....... ...... | Fried, | 21 | 3.00 | 4 | |
| Soup, barley,...... | Boiled, | 20 | 1.30 | 7 | |
| Strawberries,....... | Raw, | 12 | 2.00 | 6 | |
| Turnips,............ | Boiled, | 4 | 3.30 | 3 | |
| Veal,............... | Fried, | 25 | 4.30 | 2 | |
| Venison, ........... | Broiled. | 22 | 1.30 | 7 | |
| Wheat bread,.. .. | Baked, | 95 | 3.30 | 3 | Unbolted flour. |

